# Mycotic Popliteal Artery Aneurysm With Rapid Enlargement Post-Bypass

**DOI:** 10.7759/cureus.15746

**Published:** 2021-06-18

**Authors:** Paul Lajos, Ronald Bangiyev, Scott Safir, Thomas Weber

**Affiliations:** 1 Cardiothoracic and Vascular Surgery, University of Pittsburgh Medical Center Hamot, Erie, USA; 2 Vascular Surgery, University of Pittsburgh Medical Center Hamot, Erie, USA; 3 Vascular Surgery, Icahn School of Medicine at Mount Sinai, New York, USA; 4 Surgery, Brooklyn Veterans Affairs Medical Center, Brooklyn, USA

**Keywords:** peripheral aneurysm, mycotic aneurysm, popliteal aneurysm, infected popliteal artery aneurysm, atypical infection, incidental aneurysm, staphylococcus epidermidis

## Abstract

Popliteal artery aneurysms (PAAs) are the most common type of peripheral artery aneurysms. Mycotic aneurysms involving the popliteal artery are quite rare and can occur as either a primary *de novo *infection or a secondary infection from another site. To our knowledge, there are no previous case reports on mycotic PAA in which *Staphylococcus epidermidis* was the primary etiologic pathogen. We present the case of a 55-year-old male who presented with complaints of lower extremity pain and swelling, malaise, and low-grade temperatures for two weeks and was found to have a PAA. He underwent left femoral-popliteal bypass grafting with expanded polytetrafluoroethylene (ePTFE) graft and ligation of the aneurysm. On postoperative day 10, he experienced acute swelling and pain in his lower extremity with foot drop and was found to have rapid enlargement of his aneurysm sac on imaging. He was returned to the operating room emergently where he underwent aneurysmectomy via a posterior fossa approach. Cultures and gram staining of the aneurysm sac were consistent with *Staphylococcus epidermidis.* As noted above, this case of mycotic PAA was treated with standard vascular surgical techniques, yet it proceeded to enlarge acutely. PAAs that rapidly expand or rupture after surgical interventions may be a sign of infection.

## Introduction

Popliteal artery aneurysms (PAA) are the most common kind of peripheral aneurysms, and they typically present with acute limb ischemia due to complete thrombosis or progressive lower extremity claudication with diminished tibial runoff [1,2]. Atherosclerosis is the predominant etiology in 90% of the cases and is almost exclusively found in males with a high rate of limb loss presenting with acute ischemia, frequently bilateral and associated with aortoiliac and femoral aneurysms in 24-62% cases [1]. Rupture of PAA has an incidence rate of 2-4% in the literature and is an unusual occurrence [2].

Mycotic aneurysms have been described in virtually every arterial bed and may occur as primary or secondary infections. Primary mycotic PAAs are extremely rare with only 35 cases reported so far and none of them was due to *Staphylococcus epidermidis* [3].

## Case presentation

**Figure 1:**
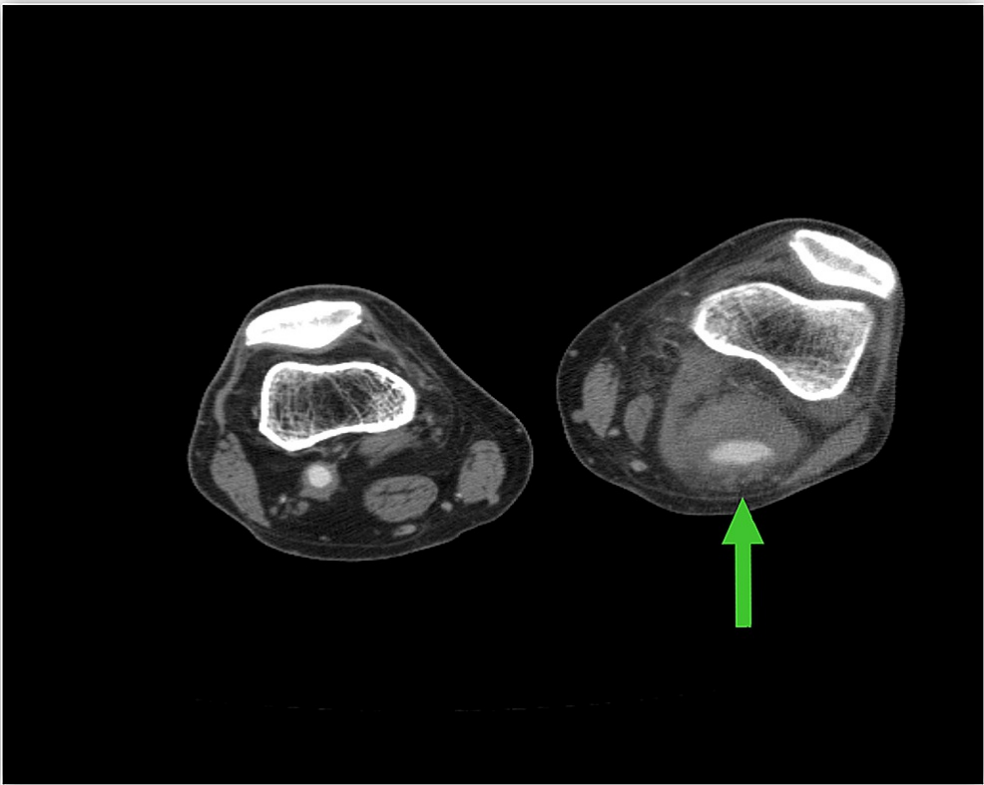
Preoperative CT scan of lower extremities The image demonstrates a left popliteal fossa mass consistent with a popliteal artery aneurysm with a patent lumen and large laminated thrombus (arrow) CT: computed tomography

A 55-year-old male presented with a two-week history of progressive left popliteal fossa pain with difficulty in walking, swollen extremity below the knee, and a history of low-grade fever. Laboratory values were unremarkable except for slightly elevated WBC and sedimentation rate. He denied any history of intravenous drug abuse (IVDA). Examination revealed a pulsating popliteal mass measuring 3 x 3 cm with a warm perfused left foot and normal pulses. CT angiogram showed a patent aneurysmal popliteal artery with thrombus measuring 3.5 x 3.5 cm (Figure 1).

**Figure 2:**
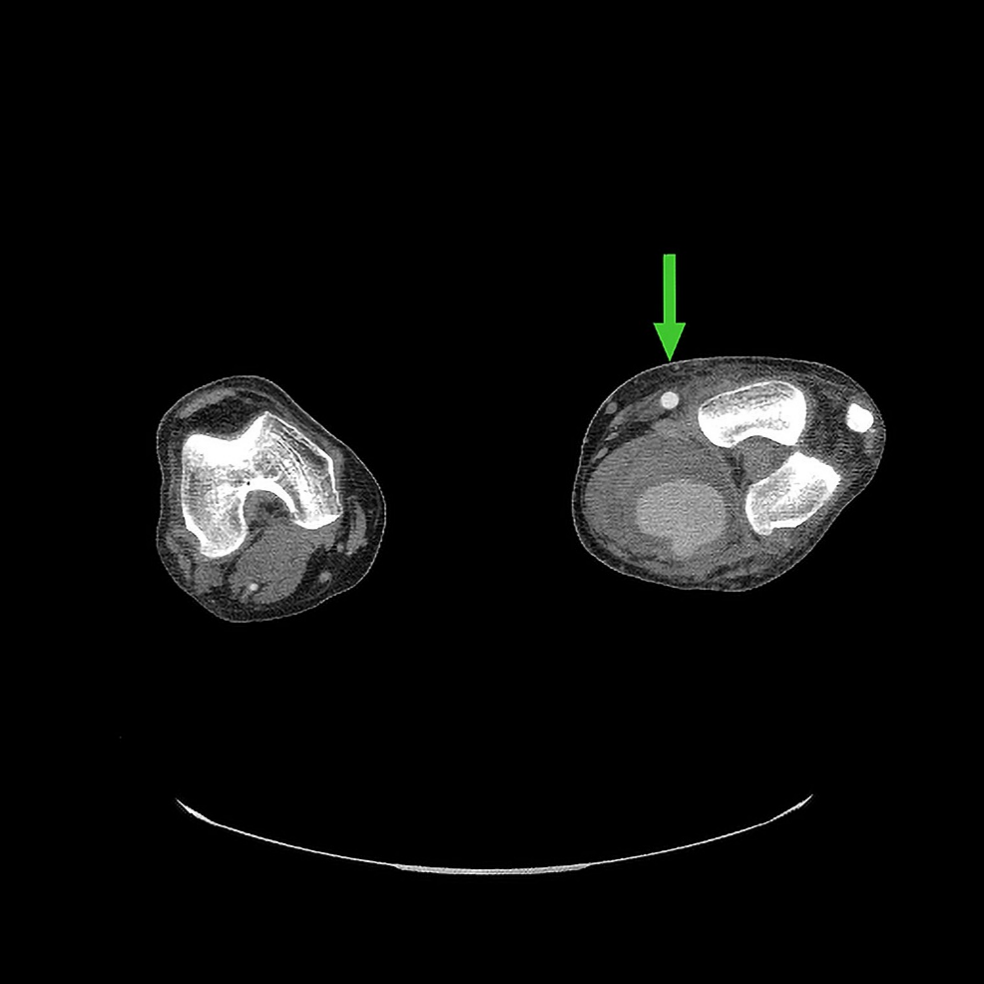
Postoperative CT scan image of left lower extremity The image shows an interval increase in the size of ligated popliteal aneurysm to 9 cm filling the left popliteal fossa. A patent bypass graft can be easily seen at 12 o’clock (arrow) immediately above the aneurysm CT: computed tomography

**Figure 3:**
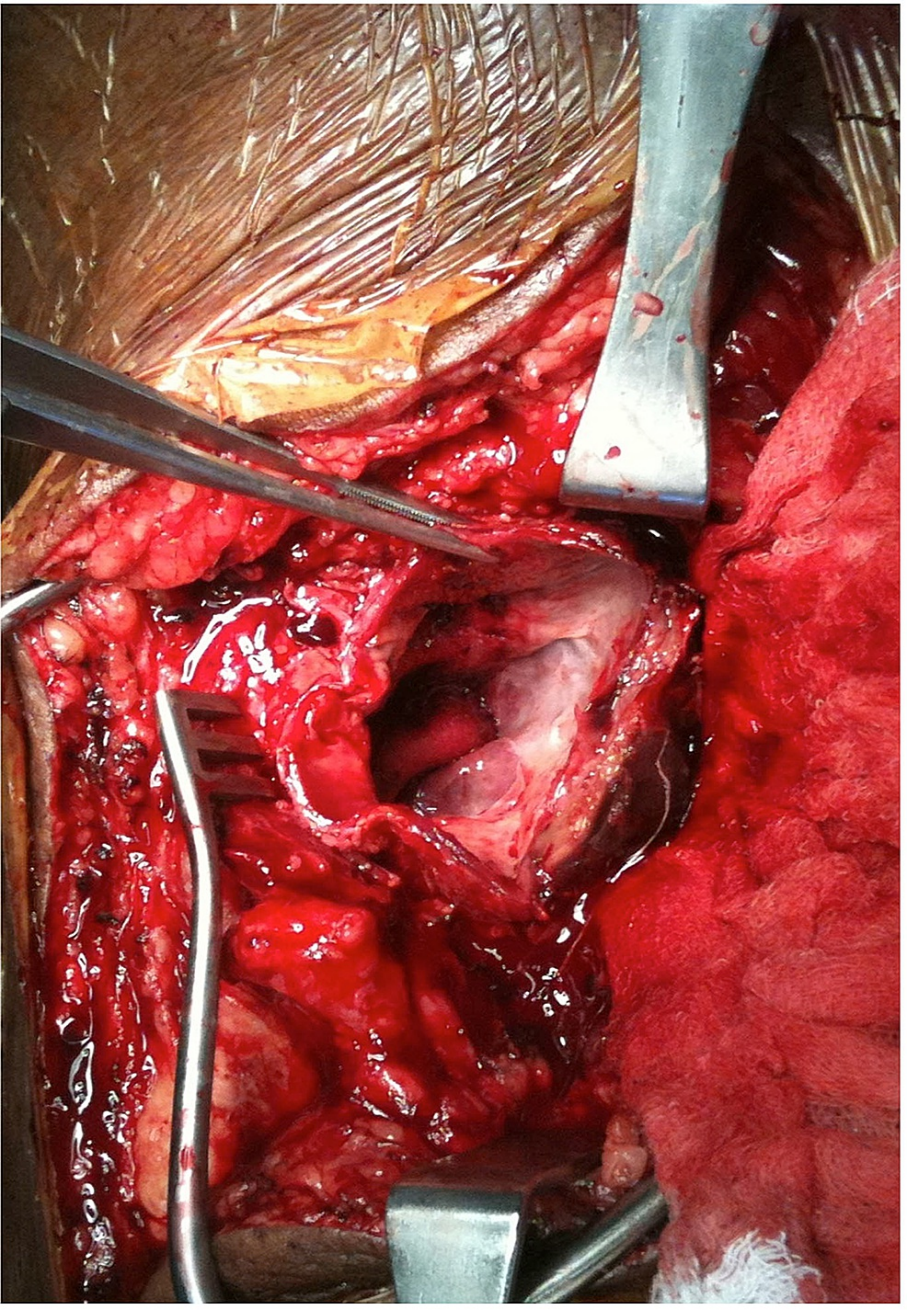
Intraoperative view of opened left popliteal artery aneurysm sac being held by surgical forceps during aneurysmectomy through posterior fossa approach This later cultured positive for *Staphylococcus epidermidis*

The patient underwent femoral-popliteal bypass grafting with expanded polytetrafluoroethylene (ePTFE) graft and ligation of aneurysm via medial approach. He tolerated the procedure until day 10 when he began to experience acute swelling and pain with foot drop. CT angiogram scan showed the expansion of PAA to 9 cm (Figure 2). He was returned emergently to the operating room for aneurysmectomy via a posterior approach (Figure 3). Intraoperative cultures and gram staining of aneurysm sac were consistent with *Staphylococcus epidermidis*. He was discharged on long-term oral antibiotics and was seen postoperatively with improvement in his symptoms.

## Discussion

Mycotic aneurysms are caused by the growth of a microorganism, typically bacterial, within the vessel wall. The term “mycotic” comes from the Greek word ‘mykes’ meaning mushroom and was first used by Osler in 1885 to describe an infected aneurysm in a patient with subacute bacterial endocarditis [4]. Endocarditis has been implicated as the etiology in roughly 16% of the cases [5]. In Salzer’s series of 28 peripheral aneurysms, the popliteal artery was the second most affected vessel and *Staphylococcus epidermidis* was seen in 7.7% of pathogens [6]. Other pathogens associated with mycotic PAA have included *Salmonella* spp., *Campylobacter fetus*, *Staphylococcus aureus*, *Listeria monocytogenes*, *Streptococcus pneumoniae*, *Streptococcus bovis*, *Streptococcus viridans*, *Brucella canis*, *Candida albicans*, and *Mycobacterium tuberculosis* [7].

Mycotic PAAs are extremely rare with a reported incidence of only 2.2% [8]. In their study of ruptured PAAs in 48 cases from 1948 to 2016, Akman et al. only noted 10 reports of mycotic PAAs [9]. Documented microbes in their series were *Salmonella* spp. (3), *Staphylococcus aureus* (2), *Escherichia coli,* *Campylobacter fetus,* *Mycobacterium bovis,* *Listeria,* and group B *Streptococcus* [9].

Overall, mycotic aneurysms infected with *Staphylococcus epidermidis* should demonstrate a generally indolent course. They may present with minimal to no clinical features of infection and are difficult to culture and diagnose. However, How et al. have reported a mycotic aneurysm in the brachial artery secondary to *Staphylococcus epidermidis* after a case of infective endocarditis that acutely enlarged and became symptomatic rapidly [10]. In their series on cryopreserved aortic reconstruction involving 71 patients, Ben Ahmed et al. showed one patient with the primary aortic infection with *Staphylococcus epidermidis* [11].

Isolated rupture of PAAs is also unusual, as presentation typically involves limb ischemia or claudication [2]. Infected PAAs as a cause of rapid expansion is virtually undocumented in the literature, and it could be attributed to underreporting, low propensity for infection due to proximity to abundant musculature and vasculature, or to the fact that culture and gram stain during surgery is not routine. The average size of ruptured PAAs at presentation was 8.2 cm (range: 2.7-13.3 cm), with a contralateral aneurysm seen in 62% of patients and an incidental abdominal aortic aneurysm seen in 33% of patients [1,2]. Pain and swelling were the most frequent complaints in 47 patients (98%), difficulty ambulating in six (13%), bruising and erythema in five (10%), and neurologic deficit in four (8%) [1,2].

Medial and posterior approaches each offer separate advantages and disadvantages. The posterior approach popularized by Szilagyi is advantageous as it provides direct access to the popliteal artery at the knee and can be useful for either aneurysmectomy or aneurysmorrhaphy [12]. It affords challenging access for conduit harvesting with limited access to the above- or below-knee artery. Medial approach allows accessibility to above or below artery for bypass and ligation and excellent conduit access for harvesting, but virtually no exposure to the popliteal fossa. Given the medial approach 10 days prior in our case, it is unlikely to have sustained a perioperative infection so quickly with no access to the aneurysm at the knee. Interestingly, Ebaugh et al. reported a 32% incidence of postoperative aneurysm enlargement when endoaneurysmorrhaphy was not used with a mean increase in the transverse diameter of 5.9 mm [13]. It was posited that this may be similar to type 2 endoleak found in endovascular aortic repairs [13]; however, none progressed as rapidly as our patient’s aneurysm.

## Conclusions

Based on our findings, the rapid enlargement of PAAs postoperatively may be a marker of infection and may warrant removal and a posterior surgical approach. The continued rapid expansion of PAA after previous bypass and ligation may be the sine qua non of an infected state.
